# Effects of Qigong, Tai Chi, acupuncture, and Tuina on cancer-related fatigue for breast cancer patients: A protocol of systematic review and meta-analysis

**DOI:** 10.1097/MD.0000000000023016

**Published:** 2020-11-06

**Authors:** Xue Li, Xueqian Wang, Lijun Song, Jiayue Tian, Xuejiao Ma, Qiyuan Mao, Hongsheng Lin, Ying Zhang

**Affiliations:** a Department of Oncology, Guang’anmen Hospital, China Academy of Chinese Medical Sciences; b School of Graduates, Beijing University of Chinese Medicine, Beijing, China.

**Keywords:** acupuncture, breast cancer, cancer-related fatigue, meta-analysis, protocol, Qigong, systematic review, Tai Chi, traditional Chinese medicine nonpharmacological interventions, Tuina

## Abstract

**Backgrounds:**

Cancer-related fatigue (CRF) is one of the most common and disabling outcomes in patients with breast cancer (BC). Traditional Chinese medicine (TCM) nonpharmacological interventions are becoming increasingly popular for cancer treatment and rehabilitation interventions. However, their efficacy and safety remain unclear and there is no systematic review or meta-analysis focusing fully on this issue. We aim to evaluate the effects of representative TCM nonpharmacological interventions, including Qigong, Tai Chi, acupuncture, and Tuina, on CRF in BC patients.

**Methods:**

Published randomized controlled trials (RCTs) that assessed the efficacy of these interventions on CRF for BC patients will be included. We will search from the following electronic databases: PubMed, Cochrane Library, EMBASE, MEDLINE, Web of Science, Scopus, PsycINFO, PSYINDEX, CINAHL, China National Knowledge Infrastructure (CNKI), WanFang Database, and Chinese Biomedical Literature Database (CBM). The primary outcomes are the improvement of CRF, which will be evaluated by the Piper Fatigue Scale (PFS), the Functional Assessment of Cancer Therapy (FACT)-Fatigue Scale, Schwartz Cancer Fatigue Scale (SCFS), the Multidimensional Fatigue Inventory (MFI). The secondary outcomes are quality of life and safety. The meta-analysis will be performed using RevMan ver 5.3(Cochrane) statistical software.

**Results:**

We will provide more practical results investigating the efficacy of Qigong, Tai Chi, acupuncture, Tuina for BC patients with CRF from several respects including the improvement of fatigue, quality of life, and safety.

**Conclusions:**

This review will generate more stronger evidence in BC patients for TCM nonpharmacological interventions, including Qigong, Tai Chi, acupuncture, Tuina, in the treatment of CRF and help to inform clinicians and policymakers.

**Ethics dissemination:**

Ethical approval is not necessary because all of the study base in our review will be based on published research. We will submit our results to a peer-reviewed journal.

**Study registration number:**

The study is priorly registered through International Platform of Registered Systematic Review and Meta-analysis Protocol on October 2, 2020 (INPLASY 2020100003)

## 1 Introduction

Breast cancer (BC) is the most common cancer among women worldwide. Fortunately, despite the increasing incidence of BC, development of cancer diagnosis and treatment technology have made year of survival extended significantly.^[1]^ For breast cancer survivors, symptom management and rehabilitation are important. Roorda study suggests that BC patients generally use of rehabilitation interventions are greater than age-matched controls.^[2]^ Cancer-related fatigue (CRF) is one of the most common and disabling outcomes reported by BC survivors during and after treatment.^[3]^ More than 80% of BC patients with standardized treatment have fatigue.^[4]^ The National Comprehensive Cancer Network (NCCN) defines CRF as a distressing, persistent, subjective sense of physical, emotional, and/or cognitive tiredness or exhaustion related to cancer or cancer treatment that is not proportional to recent activity and interferes with usual functioning.^[5]^ CRF is exacerbated by higher rates of depression,^[6,7]^ sleep disturbance,^[8,9]^ and pain,^[6,10]^ it is highly bothersome, interferes with daily activities, and limiting overall quality of life. Besides, CRF can persist for up to 10 years after end of treatment.^[11]^ It is increasingly demanding to improve quality of life.

At present, common prevention and treatment agents for CRF includes psychostimulants, corticosteroids, and erythropoietin. However, they are not extensively used in clinical because of their potential serious adverse effects and insignificant efficacy. Other pharmacologic interventions remain investigational, and some have even been identified as ineffective.^[5]^ Hence, there is growing interest in nonpharmacological interventions that are safe and few side effects. Some clinical studies have shown that nonpharmacological interventions, such as exercise,^[12,13]^ nutrition therapy,^[14,15]^ and psychological interventions^[16,17]^ are effective in relieving fatigue for BC patients. The Oncology Nursing Society considers nonpharmacological interventions as a promising intervention for CRF,^[18]^ and similar recommendations have been made by the NCCN.^[5]^ Under the circumstance, nonpharmacological interventions has been gaining more attention in CRF treatment for BC patients around the world.

In China, TCM nonpharmacological interventions have been widely applied in management of fatigue. The most commonly used and internationally recognized methods include Qigong, Tai Chi, acupuncture, and Tuina.^[19–21]^ According to the TCM theory, CRF is one kind of consumptive disease due to disorder of viscera function and insufficiency of Qi and blood, Yin and Yang. Qigong, Tai Chi, acupuncture, and Tuina have the function of regulating visceral function, reinforcing Qi and activating blood, and restoring the balance of Yin and Yang. In addition, numerous clinical reports from various sources have shown that Qigong,^[22]^ Tai Chi,^[23]^ acupuncture,^[24]^ and Tuina^[25]^ are effective for CRF. They can also alleviate depression,^[26]^ sleep disturbance,^[27]^ and pain.^[28]^ And these interventions are less likely to have side effects.

Previous studies have proved the validity of TCM nonpharmacological interventions on fatigue.^[29–31]^ But there is no consensus on CRF in breast cancer. Though some researches applying Qigong, Tai Chi, acupuncture, and Tuina in BC patients with CRF have described positive results, others have reported mixed evidence.^[32]^ Hence, our research team is planning a systematic review and meta-analysis investigating the efficacy of CRF managements through representative TCM nonpharmacological interventions, including Qigong, Tai Chi, acupuncture, and Tuina, to identify the treatment with the better efficacy for practical consideration for BC patients.

## 2 Methods

### 2.1 Eligibility criteria

#### 2.1.1 Types of studies

Randomized controlled trials (RCTs) will be included without restriction of publication type or language. Studies should be available in full papers and peer-reviewed.

#### 2.1.2 Types of participants

Breast cancer patients suffering from CRF with the following conditions will be included: firstly, age, sex, race, education status, and types of treatment are not restricted. Secondly, definite pathological diagnosis of breast cancer without restrictions related to type and stage. And finally, breast cancer patients should conform to the diagnosis standards of CRF, which based on International Statistical Classification of Diseases and Related Health Problems, 10th revision (ICD-10).

#### 2.1.3 Types of interventions and comparators

Approaches including Qigong, Tai Chi, acupuncture, and Tuina alone or in combination, will be reviewed. The control group could be placebo, blank control, standard care, and other body-based practices such as exercise techniques, yoga.

#### 2.1.4 Types of outcome measures

##### 2.1.4.1 Primary outcomes

The primary outcomes are certain common scales which reflect fatigue severity. We considered the following scales: Piper Fatigue Scale (PFS); the Functional Assessment of Cancer Therapy (FACT)-Fatigue Scale; Schwartz Cancer Fatigue Scale (SCFS); the Multidimensional Fatigue Inventory (MFI).

##### 2.1.4.2 Secondary outcomes

The secondary outcome measures are any quality of life, adverse events.

### 2.2 Information sources and search strategy

#### 2.2.1 Electronic searches

RCTS are being searched in the following electronic databases without language and publication date restrictions: PubMed, Cochrane Library, EMBASE, MEDLINE, Web of Science, Scopus, PsycINFO, PSYINDEX, CINAHL, China National Knowledge Infrastructure (CNKI), WanFang Database, and Chinese Biomedical Literature Database (CBM) until March 2020. Search terms are related to breast cancer, cancer related fatigue, Qigong, Tai Chi, acupuncture, and Tuina. The details of the PubMed database search strategy are shown in Table 1, and similar search strategies will be adopted for other databases.

**Table 1:** Search strategy for PubMed.

No.	Search items
#1	Breast Carcinoma[Mesh Terms]
#2	breast neoplasms[Title/Abstract]
#3	breast carcinoma[Title/Abstract]
#4	breast carcinomas[Title/Abstract]
#5	breast neoplasms[Title/Abstract]
#6	breast tumor[Title/Abstract]
#7	breast tumour[Title/Abstract]
#8	breast cancer[Title/Abstract]
#9	breast cancers[Title/Abstract]
#10	#1 OR #2 OR #3 OR #4 OR #5 OR #6 OR #7 #8 OR #9
#11	fatigue[Mesh Terms]
#12	cancer-related fatigue[Mesh Terms]
#13	fatigue[Title/Abstract]
#14	cancer-related fatigue[Title/Abstract
#15	#11 OR #12 OR #13 OR #14
#16	Qigong[Mesh Terms]
#17	qigong[Title/Abstract]
#18	Baduanjin[Title/Abstract]
#19	Wuqinxi[Title/Abstract]
#20	Tai Chi[Mesh Terms]
#21	Tai Chi[Title/Abstract]
#22	Taiji[Title/Abstract]
#23	Taijiquan[Title/Abstract]
#24	Tai Chi Chuan [Title/Abstract]
#25	Acupuncture[Mesh Terms]
#26	Acupressure[Mesh Terms]
#27	acupuncture[Title/Abstract]
#28	acupressure[Title/Abstract]
#29	acupoint[Title/Abstract]
#30	moxibustion[Title/Abstract]
#31	Tuina[Mesh Terms]
#32	Massage[Title/Abstract]
#33	tuina[Title/Abstract]
#34	massage[Title/Abstract]
#35	#16 OR #17 OR #18 OR #19 OR #20 OR #21 OR #22 OR #23 OR #24 OR #25 OR #26 OR #27 OR #28 OR #29 OR #30 OR #31 OR #32 OR #34 OR #35
#36	randomized controlled trial [Publication Type]
#37	controlled clinical trial [Publication Type]
#38	randomized [Title/Abstract]
#39	randomly [Title/Abstract]
#40	trial [Title/Abstract]
#41	groups [Title/Abstract]
#42	placebo[Title/Abstract]
#43	#36 OR #37 OR #38 OR #39 OR #40 OR #41 OR #42
#44	#10 AND #15 AND #35 AND #43

#### 2.2.2 Searching other resources

Manual searches will include reviewing reference lists of identified studies, relevant reviews, meta-analyses, and journals that have published the most relevant research articles or reviews. Meanwhile, grey literature will be searched. The ongoing RCTS will be searched the WHO International Clinical Trial Registry Platform (ICTRP) and its Registry Network, and we will contact corresponding authors to identify extra studies if necessary.

### 2.3 Study selection

**Figure 1:**
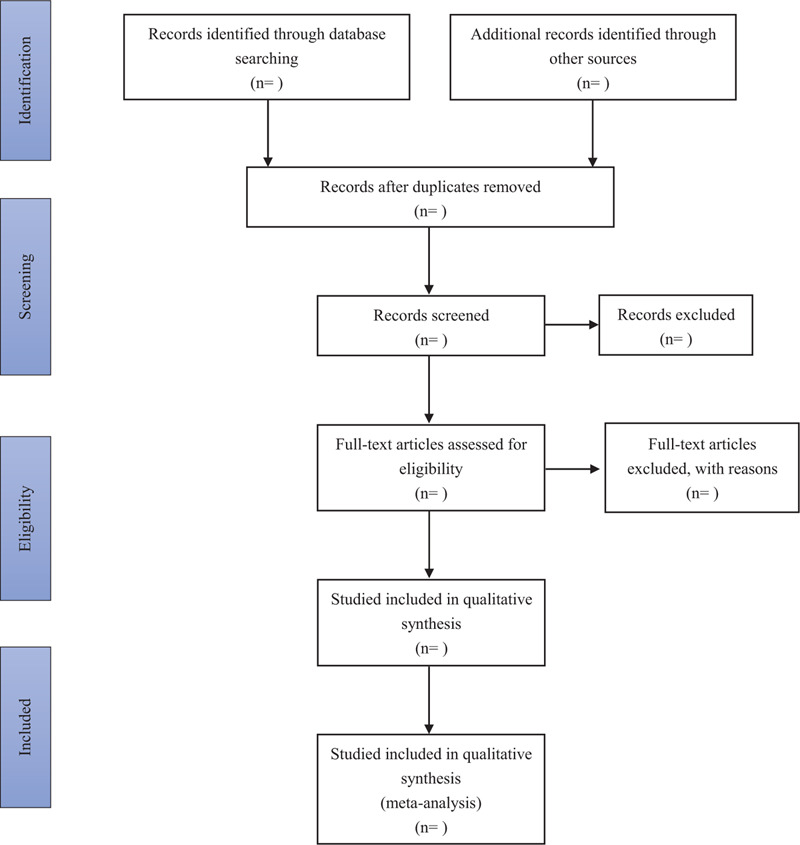
Flow diagram of study selection.

First of all, the titles and abstracts of researches will be independently examined by 2 review authors to make a preliminary selection of potential trails according to our pre-determined eligibility criteria. Second, full text of all preliminary selective trials will be downloaded to make sure eligible trials. The unclear information or missing data will be replenished by contacting with the authors. Discrepancies between 2 reviewers will be resolved through discussion or be arbitrated of a third reviewer. A Preferred Reporting Items for Systematic Reviews and Meta-Analyses (PRISMA) flow chart (Fig. 1) will be used to describe the entire selection process.

### 2.4 Date extraction

Information from eligible RCTs will be extracted independently by 2 authors. Extracted information will include: published materials: title, authors, publication date, country, study design, type of control, the number of study groups, and center; the population: sample size (treatment/control), age, sex, race ratio; the intervention: duration, frequency, intensity; outcomes and others: scale tools, evaluation time, outcome details, adverse events and complications, quality of life. Disagreements will be resolved through team discussion.

### 2.5 Risk of bias assessment

Two reviewers will independently assess the risk of study bias using the Cochrane Collaboration tool, which consists of the following 6 items: random sequence generation, allocation concealment, participant and personnel blinding, outcome assessment blinding, incomplete outcome data, selective reporting, and other source of bias. The quality of the reporting will be categorized into 3 levels: low, unclear, and high risk of bias. Any disagreement will be decided by 3rd reviewer.

### 2.6 Dealing with missing data

As for the lacking data, insufficient or vague, we will contact the corresponding authors by email or telephone to obtain missing information. If fail, we will only conduct analysis based on available data and potential impact of the incomplete data will be analyzed and reported in the summary results.

### 2.7 Data synthesis and analysis

Meta-analysis will be performed using RevMan ver 5.3 (Cochrane, Oxford, England) statistical software. Relative risk (RR) will be used when the result is dichotomous variables and 95% confidence intervals (CI). For continuous variables, we will use standardized mean difference (SMD) and 95% CI. Chi-squared test and *I*^2^ statistic will be used to confirm the heterogeneity. The former checks for heterogeneity, while the latter reflects the degree of heterogeneity through a specific value. *I*^2^ of 25%, 50%, and 75%, respectively, indicated low, medium, and high heterogeneity. If *I*^2^ is >50%, there is considerable heterogeneity between studies, so a subgroup analysis will be performed to investigate the potential causes.

### 2.8 Measures for publication bias

When included studies are >10, funnel plot will be used to identify the publication bias. Begg and Egger tests will be utilized to evaluate funnel plot symmetry.

### 2.9 Evaluation of the level of evidence

Two reviewers will independently evaluate the level of evidence for outcomes according to the Grading of Recommendations Assessment, Development and Evaluation (GRADE). There are 4 possible levels: very low, low, moderate, or high. The level of evidence is determined by the seriousness of 5 factors: study limitations, inconsistency, imprecision, indirectness, and publication bias.

### 2.10 Subgroup analysis

Considering significant heterogeneity, we plan to carry out a subgroup analysis. The following items will be considered: age, and race of patients, types and stage of breast cancer, course of the intervention.

### 2.11 Sensitivity analysis

Sensitivity analysis will be conducted to eliminate the efficacy of low quality studies, provided there is significant heterogeneity after robust subgroup analysis. The meta-analysis will be repeated after low-quality studies are removed. We will compare the results of the 2 meta-analyses and then decide whether to exclude low-quality studies based on evidence strength, sample size, and influence on the pooled estimate. Nonetheless, sensitivity analysis will not be performed if there is a high risk of bias in all included studies.

## 3 Discussion

Due to their efficacy and few adverse effects, TCM nonpharmacological interventions have been widely recognized and used in terms of improving the quality of life and symptoms of cancer patients. For breast cancer patients, fatigue, as a most common and bothersome symptom, frequently cooccurs with other symptoms. Consequently, it is especially crucial to determine the effective treatments with few adverse effects that can improve fatigue in breast cancer patients. TCM nonpharmacological interventions have great potential in this respect.

Now, some possible biological mechanisms for Tai chi, Qigong, acupuncture, and Tuina of breast cancer related fatigue have been suggested, such as a decrease of inflammatory cytokines,^[33]^ an increase of T-lymphocytes^[34]^ and beta-endorphins,^[35]^ and improving immune function.^[36]^ But clinical evidence-based researches of these interventions are limited. In order to provide more objective evidence of Tai chi, Qigong, acupuncture, and Tuina for breast cancer related fatigue, a high-quality systematic review and meta-analysis is necessary.

There are some limitations to this study. First, we have developed strict inclusion criteria to ensure the quality of research. However, it may lead to limited number of studies. Second, we will only include studies published in English or Chinese due to the language barrier, which might cause publication bias to some extent. Finally, we only evaluated the most widely used and representative TCM nonpharmacological interventions, which may have some limitations in proving the efficacy of TCM nonpharmacological intervention for breast cancer related fatigue. In spite of these limitations, this study will not only help establish a better approach to prevent and treat CRF in breast cancer patients, but also might have the potential to improve quality of life for breast cancer patients worldwide.

## Author contributions

**Conceptualization:** Xue Li, Xueqian Wang.

**Data curation:** Lijun Song, Jiayue Tian.

**Investigation:** Xuejiao Ma, Qiyuan Mao.

**Methodology:** Lijun Song, Jiayue Tian, Qiyuan Mao.

**Supervision:** Ying Zhang, Hongsheng Lin.

**Writing – original draft:** Xue Li, Xueqian Wang.

**Writing – review & editing:** Xue Li.
